# Synthesis and properties of shape memory polyurethanes generated from schiff-base chain extender containing benzoyl and pyridyl rings

**DOI:** 10.1080/15685551.2018.1450467

**Published:** 2018-03-19

**Authors:** Neng-Chiao Weng, Chih-Fu Wu, Wen-Chin Tsen, Cheng-Lung Wu, Maw-Cherng Suen

**Affiliations:** a The Graduate Institute of Design Science, Tatung University, Taipei, Taiwan, R.O.C.; b Department of Fashion Styling & Design, LEE-MING Institute of Technology, Taipei, Taiwan, R.O.C.; c Department of Fashion Business Administration, LEE-MING Institute of Technology, Taipei, Taiwan, R.O.C.; d Department of Materials Science and Engineering, National Taiwan University of Science and Technology, Taipei, Taiwan, R.O.C.

**Keywords:** Polyurethanes, hydrogen bonding, thermal properties, shape memory

## Abstract

In this study, 4,4′-diphenylmethane diisocyanate and polytetramethylene glycol were used to prepare a prepolymer; N,N′-bis(4-hydroxybenzylidene)-2,6-diaminopyridine (BHBP) was used as a chain extender; and these elements were combined to prepare a novel polyurethane, BHBP/PU. Gel permeation chromatography revealed that the molecular weight of the BHBP/PU samples increased as the BHBP content was increased. Fourier transform infrared spectroscopy demonstrated that high BHBP content facilitated strong hydrogen bonding in the samples. Differential thermogravimetry indicated that the initial decomposition temperature of BHBP/PU-3 was approximately 10 °C higher than that of BHBP/PU-1. Differential scanning calorimetry and dynamic mechanical analysis revealed that increasing the BHBP content substantially increased both the glass transition and dynamic glass transition temperatures of the BHBP/PU samples. The tensile strengths of BHBP/PU-1, BHBP/PU-2, and BHBP/PU-3 were 7.7, 10.9, and 21.6 MPa, respectively, with corresponding Young’s moduli of 0.7, 1.9, and 3.3 MPa. These results demonstrated that both the tensile strength and Young’s modulus of the BHBP/PU samples increased as the BHBP content was increased. Moreover, the BHBP/PU samples exhibited excellent shape recovery of >90%.

## 1. Introduction

Polyurethanes (PUs) were first discovered by Otto Bayer in the 1940s [1]. Since their discovery, PUs have increasingly attracted attention because of their wide range of industrial applications such as in shipbuilding, automobiles, footwear, and construction [2,3]. PUs are usually obtained from the exothermic reaction between polyisocyanates and polyols [4], and ingredients such as long chain polyols, diisocyanate, and short chain extenders and employed to control their thermoplastic properties [5]. PUs can be synthesized to form elastomers with various properties through various ratios of soft and hard segments. Their unique properties have led to widespread research on applications such as adhesives, sealants, textiles, medical equipment, and waterproof coating [6–10].

Shape-memory polymers (SMPs) have the ability to ‘remember’ their shape and recover to this permanent shape after a deformation has been induced by an external stimulus [11]. Shape-memory effects may be triggered by stimuli such as temperature, light, and pH [5]. For example, in thermally induced SMPs, the thermoreversible phase that serves as a ‘switch’ is the transition between the glass transition temperature (T_g_) and the melting point (T_m_) [12]. Light-induced SMPs achieve the reversible process when stimulated by molecular switches [13]. In addition, other shape-memory effects such as moisture sensitivity can act as molecular switches [14,15]. Compared with shape-memory alloys, SMPs have advantages such as extremely flexible processing conditions, brightness, weight, and material design [11,16–19]. Papers have reported that pyridine-containing urethanes can be used as a shape-memory material and confirmed that the movement of pyridine-containing polymer chains is influenced by the dissociation of hydrogen bonds, leading to the strain recovery of PUs. In addition, one study examined the moisture absorption of pyridyl units and concluded that moisture sensitivity can be a molecular switch for pyridine-containing PUs [20].

Some recent studies have investigated the thermoreversible noncovalent bonds of SMPs. In one study [21], the authors synthesized supramolecular PU networks containing pyridine moieties and observed hydrogen bonding between the nitrogen atoms in the pyridine and N–H bonds. The effect of ambient temperature on these hydrogen bonds enabled 90% shape memory of the PUs. The pyridine-containing supramolecular PUs synthesized in [22] used the nitrogen atoms in the pyridine to facilitate hydrogen bonding with urethane segments. These hydrogen bonds served as the molecular switch and enabled the shape-memory effect of the PUs.

Some other studies have introduced highly functional pyridine rings to polymers through chemical synthesis. For example, the authors of [23] used 2,6- pyridinedicarboxamide as the chain extender to synthesize linear and crosslinked PUs. Thermogravimetric analysis (TGA) revealed that the crosslinked PUs had substantially improved thermal stability and mechanical properties. By contrast, another study [24] used 1,6-hexamethylene diisocyanate with 2,3-dihydroxy-pyridine or 2-amino-3-hydroxy-pyridine as the chain extender to synthesize PUs. The results revealed that the fixed polymer segment did not contribute to the segmental relaxation, resulting in dielectric strength decreases with the presence of crosslinks.

In the present study, 2,6-diaminopyridine and 4-hydroxybenzaldehyde were synthesized to obtain the pyridine-containing chain extender N,N′-bis(4-hydroxybenzylidene)-2,6-diaminopyridine (BHBP). Both the nitrogen atoms of pyridyl and imine groups in BHBP units can form hydrogen bondings with N-H groups in the PU units theoretically. The above hydrogen bonding favors the shape memory effect of BHBP/PU polymers. In addition the phenylene groups of BHBP units can enhance the thermal resistance and mechanical strength of the polymers. Due to this special structure of BHBP, we use it as a chain extender in this work. Subsequently, polytetramethylene glycol (PTMG) and 4,4’-diphenylmethane diisocyanate (MDI) were reacted to obtain the prepolymer of the target PU. The BHBP chain extender was added to the prepolymer to form BHBP/PU polymers. Altering the soft and hard segments of the PTMG and the BHBP content, this study investigated the thermal properties, mechanical properties, shape recovery, and moisture sensitivity of the BHBP/PU polymers.

## 2. Experimental

### 2.1. Materials and synthesis of BHBP/PUs

MDI was purchased from Sigma-Aldrich. PTMG (Mw = 1000) was obtained from Polyscience (U.S.A.). N,N-dimethylacetamide (DMAc) was obtained from Mallinckrodt Chemicals. BHBP was synthesized using the method described in [25]. All the raw materials used were synthetic grade.

**Figure 1.**
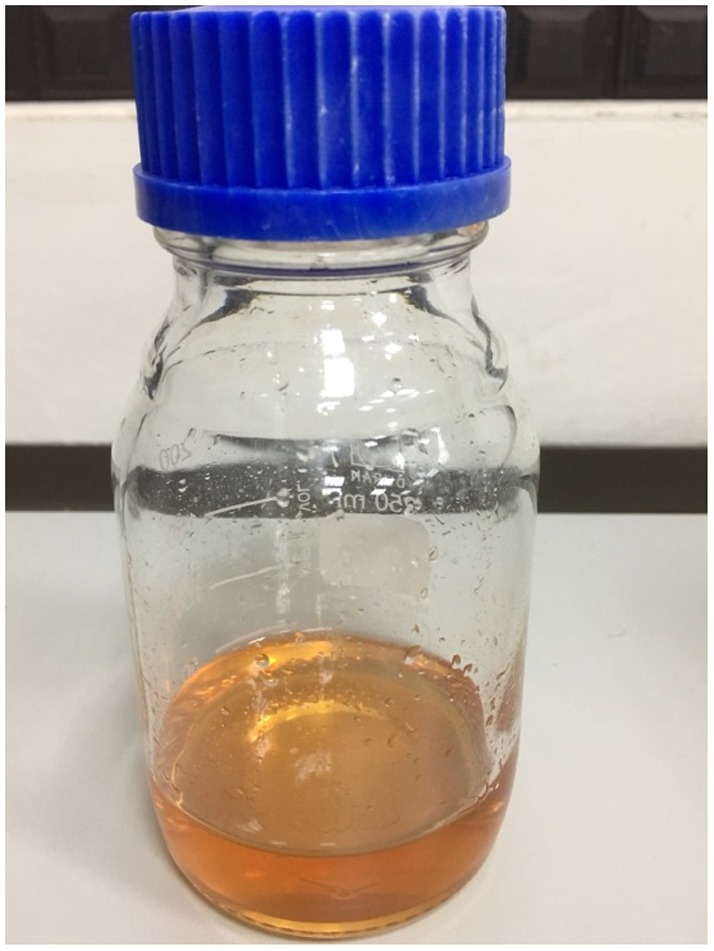
Picture of one BHBP/PU solution.

**Scheme 1.**
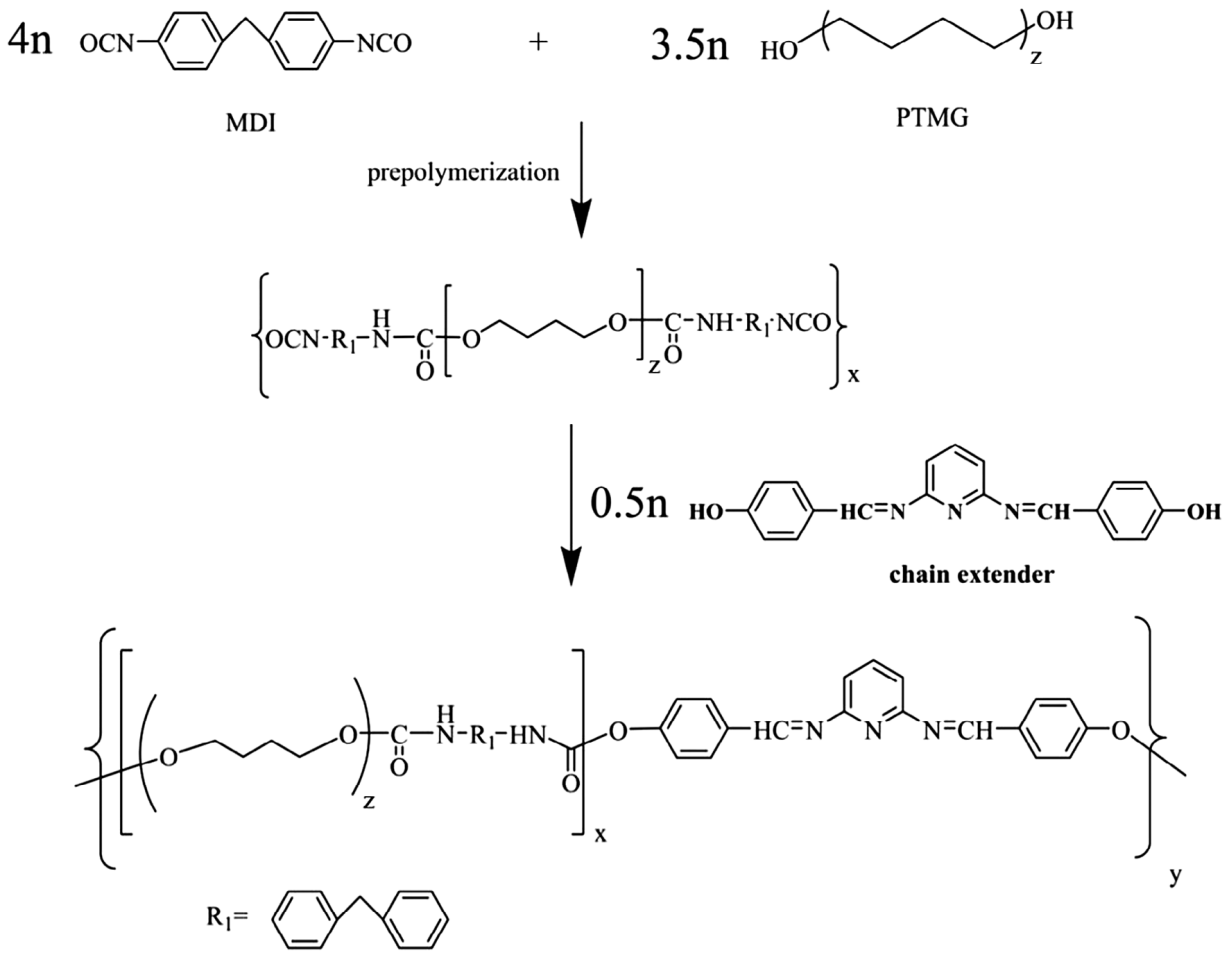
Representative BHBP/PUs reaction formula.

The prepolymer in this experiment consisted of aromatic MDI as the hard segment and PTMG as the soft segment, whereas the chain extender was BHBP. First, MDI, PTMG, and DMAc were placed in a 500-mL three-necked reaction flask. After being heated to 80 °C in nitrogen atmosphere, the flask was placed in a mechanical stirrer operated at 200 rpm. The prepolymer was formed after 2 h of stirring. Subsequently, BHBP was dissolved in DMAc before being added to the prepolymer and the mixture was left to react for 2 h (Scheme 1). The BHBP/PU solutions were poured into serum bottles (Figure 1) and stored in a refrigerator for one day to eliminate possible bubbles in the viscous BHBP/PU solutions. Subsequently, the BHBP/PU solutions were poured into polytetrafluoroethylene (Teflon) molds and placed in a heating oven at 80 °C for 8 h to remove most DMAC solvent gradually. The heating temperature at 80 °C that far below the boil point (165 °C) of DMAc can avoid causing bubbles during the mild evaporation of DMAc. Then, the crude BHBP/PU films were heated in the oven at 100 °C under dynamic vacuum for 8 h to remove residual DMAc completely. Finally, the obtained BHBP/PU thin films were removed from the Teflon molds, designated as BHBP/PU-1, BHBP/PU-2 and BHBP/PU-3. All the raw materials used for preparing BHBP/PU solutions were synthetic grade and no free NCO groups were detected (indicating complete reaction of monomers) by FT-IR spectroscopy as discussed in the following ‘2.2 Gel permeation chromatography’ subsection. Thus the BHBP/PU polymers could be directly used in the characterization and performance tests without prior purification. Table 1 shows the chemical formulae and hard and soft segment contents of each BHBP/PU. The hard and soft segment contents were obtained using the following equations:

**Table 1.** Formula of the BHBP/PUs.

Designation	MDI (moles)	PTMG (moles)	BHBP (moles)	Hard segment (wt%)	Soft segment (wt%)
BHBP/PU-1	4	3.50	0.50	24.87	75.13
BHBP/PU-2	4	3.25	0.75	27.58	72.42
BHBP/PU-3	4	3.00	1.00	30.51	69.49

Hard segment content (wt%) = $\frac{W_{\mathrm{MDI}}+W_{\mathrm{BHBP}}}{W_{\mathrm{MDI}}+W_{\mathrm{PTMG}}+W_{\mathrm{BHBP}}}\times \;100\%$ (2.1)

Soft segment content (wt%) = 100% − Hard segment content (wt%) (2.2)

• *W*_*MDI*_: molar weight of MDI
• *W*_*BHBP*_: molar weight of BHBP
• *W*_*PTMG*_: molar weight of PTMG

#### 2.1.1. Gel permeation chromatography (GPC)

GPC was performed using an Analytical Scientific Instruments Model 500. A refractive index detector (Schambeck RI2000), two Jordi divinylbenzene columns in a series of mixed beds, and a 10^4^-Å bed at 30 °C were used to measure the molecular weight distribution relative to the polystyrene standard. The eight standard samples for calibration had a molecular range of 3.42 × 10^3^ to 2.57 × 10^6^. The carrier solvent was tetrahydrofuran, and the flow rate used was 1 mL/min.

#### 2.1.2. Fourier transform infrared spectroscopy (FT-IR)

FT-IR was performed using a PerkinElmer spectrometer (Model Spectrum One) with a scanning range of 4000–650 cm^−1^ and a resolution of 2 cm^−1^. The values of 16 scans were averaged.

#### 2.1.3. Thermogravimetric analysis (TGA)

TGA was conducted using a PerkinElmer analyzer (Model Pyris 1). The BHBP/PU samples were used in masses of 5–8 mg. The initial temperature under nitrogen atmosphere was set at 50 °C and was increased to 700 °C using a heating rate of 10 °C/min.

#### 2.1.4. Differential scanning calorimetry (DSC)

DSC was performed using a PerkinElmer calorimeter (model Jade). The BHBP/PU samples were sealed on a covered aluminum plate and scanned at 10 °C/min in nitrogen atmosphere of −80 to 50 °C. T_g_ was determined by the midpoint of the slope of the DSC curve. All tested BHBP/PU samples were used in masses of 5–8 mg.

#### 2.1.5. Dynamic mechanical analysis (DMA)

DMA was performed using a Seiko dynamic mechanical spectrometer (Model SII Muse, DMS6100). The BHBP/PU films had dimensions of 20 × 5 × 0.2 mm^3^ (L × W × H). The stress test conducted between −50 and 50 °C had the following parameters: 1-Hz frequency, 5-μm amplitude, and 3 °C/min heating rate.

#### 2.1.6. Universal testing machine

Destructive physical analysis was conducted using BHBP/PU thin films with dimensions of 45 × 8 × 0.2 mm^3^ (L × W × H) under the ASTM D-638 standard. A universal testing machine was used to measure the tensile strength and elongation at break of the BHBP/PU samples and obtain destructive test data.

#### 2.1.7. Shape recovery test

A cyclic tensile loading test was performed using a test machine with a temperature control chamber. The samples consisted of thin films with dimensions of 40 × 5.0 × 0.5 mm^3^ (L × W × H). The samples were placed in the chamber at under room temperature and heated to 65 °C using a heating rate of 3 °C/min. After 10 min, stress was applied using a crosshead speed of 10 mm/min at 65 °C. The strain measured in the 100% stretched samples was denoted *ε*_*m*_.

Subsequently, the samples were rapidly cooled to 25 °C, and the strain measured after 10 min of stress relief at 25 °C was denoted *ε*_*u*_. Finally, the samples were heated to 85 °C using a heating rate of 3 °C/min, and the strain measured 10 min later was denoted *ε*_*p*_. The following equations were then used to assess each sample’s shape recovery:

Shape recovery (%), $R_{r}=\frac{\left(\varepsilon_{m}-\varepsilon_{p}\right)}{\varepsilon_{m}}\times 100$ (2.3)

Shape fixity (%), $R_{f}=\frac{\varepsilon_{u}}{\varepsilon_{m}}\times 100$ (2.4)

## 3. Results and discussion

### 3.1. GPC analyses

**Figure 2.**
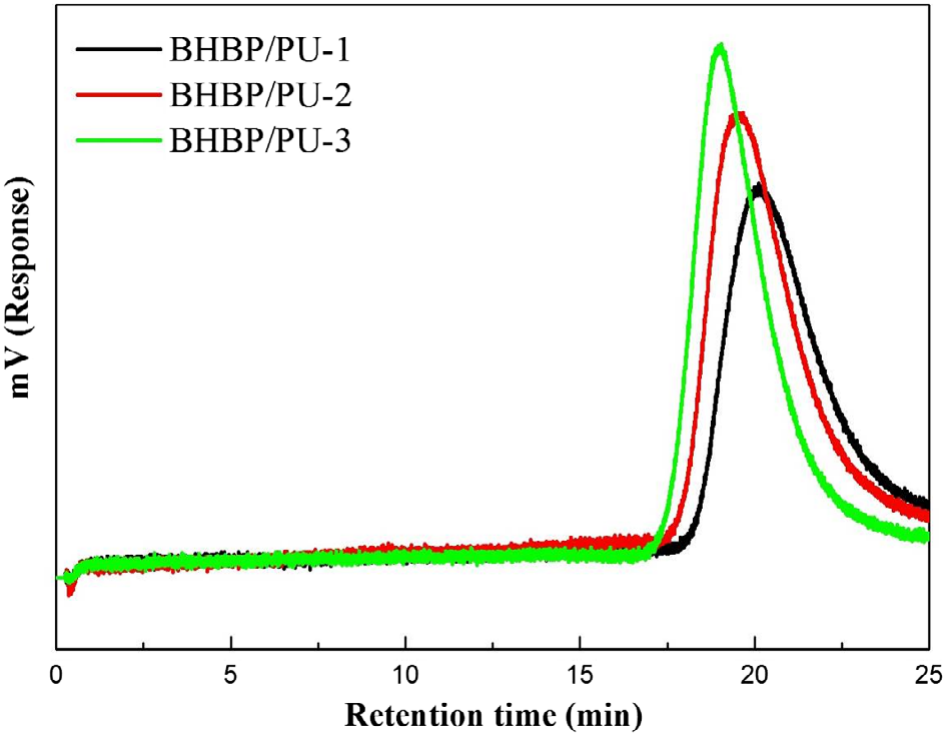
GPC curves of BHBP/PUs.

Figure 2 presents the GPC chromatograms of BHBP/PU samples with various proportions of BHBP. The curves of all three samples were unimodal, indicating no residual raw materials in the samples. Figure 2 displays both the weight-averaged molecular weight (M_w_) and number-averaged molecular weight (M_n_) of each BHBP/PU sample. The samples with higher BHBP content had higher molecular weight (Table 2). Molecular weight was depicted by the fraction M_w_/M_n_. In this study, the fraction ranged between 1.4 and 1.6. As the BHBP content was increased, the BHBP/PU molecular distribution became narrower.

### 3.2. FT-IR spectra

**Figure 3.**
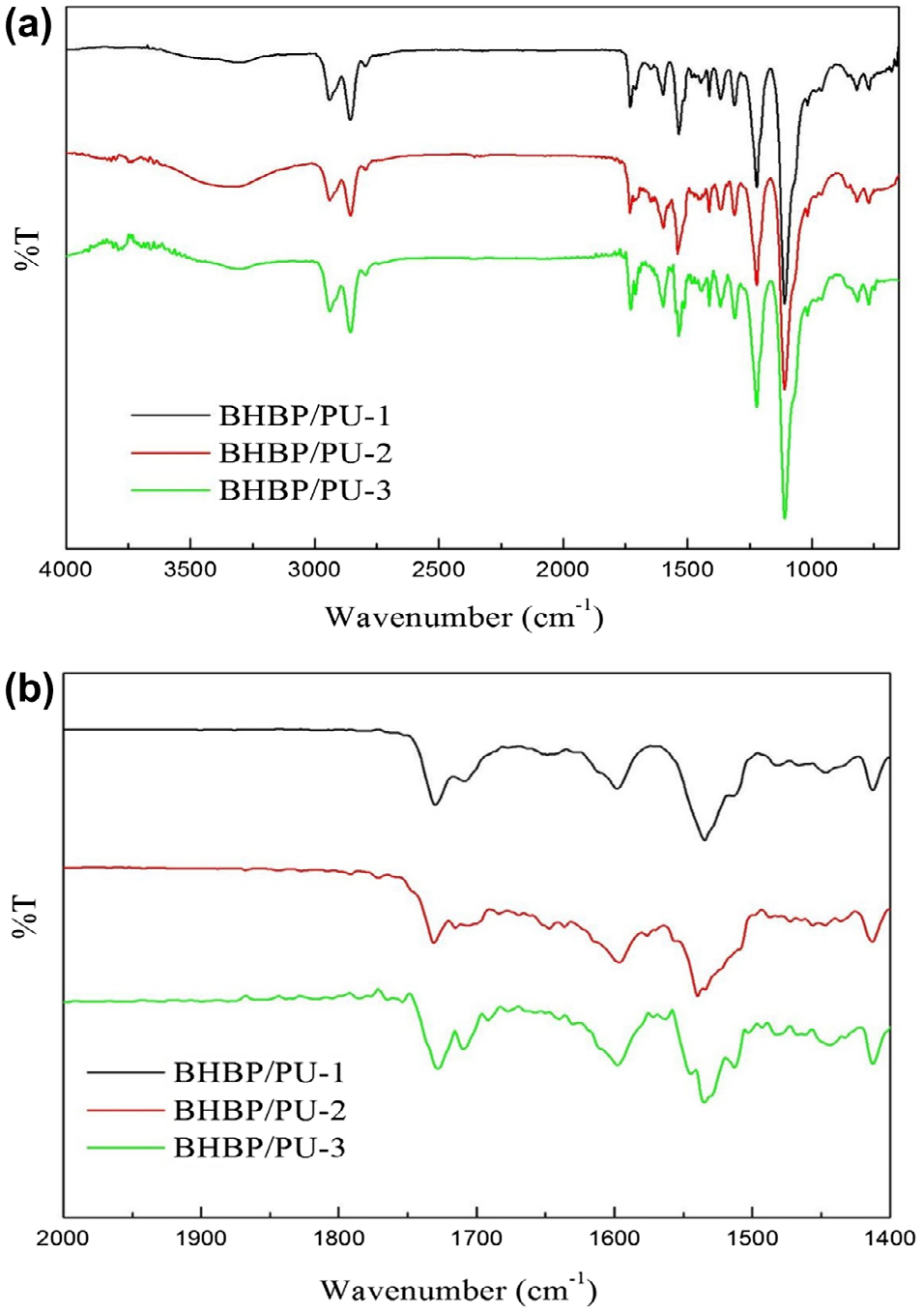
FT-IR spectra of the BHBP/PUs at the wavenumber range of (a) 4000–650 cm^−1^; (b) 2000–1400 cm^−1^.

Figure 3(a) presents the FT-IR spectra of the BHBP/PU samples between the wavelengths 4000 and 650 cm^−1^. Five major peaks were observed in all samples: a N–H stretching vibration peak at approximately 3306 cm^−1^; a CH_2_ stretching vibration peak between 2939 and 2854 cm^−1^; a C=O stretching vibration peak at approximately 1729 cm^−1^; a N–H bending vibration peak at approximately 1535 cm^−1^; and a C–O–C stretching vibration peak at approximately 1017 cm^−1^. The FT-IR results demonstrated the formation of PU structures and no free N=C=O groups, the peak of which would have appeared at 2275 and 2240 cm^−1^. During synthesis, the NCO end of the prepolymer had completely reacted with BHBP.

Despite the similarity of the three spectra corresponding to the BHBP/PU samples with various BHBP content, slight variances were observed. Figure 3(b) illustrates that the wave number of the absorption peak ranged between 2000 and 1400 cm^−1^. Free C=O bonds and hydrogen bonds were observed at 1729 and 1708 cm^−1^, respectively. As the BHBP content was increased, the peak corresponding to C=O bonds shifted from 1729 to 1708 cm^−1^ and substantially increased in intensity because of the influence of hydrogen bonds. As the BHBP content was increased, strong hydrogen bonds were formed between the C=O and N–H bonds and between the pyridine rings and N–H bonds in the amine ester groups, forming a complex supramolecular network structure in the BHBP/PU samples. In addition, a C–N–C stretching vibration peak was observed at 1598 cm^−1^ and intensified as the BHBP content was increased.

**Table 2.** GPC result of BHBP/PUs.

Sample	Retention time of the peak (min)	$\bar{\mathrm{Mn}}$ (× 10^4^)	$\bar{\mathrm{Mw}}$ (× 10^4^)	$\bar{\mathrm{Mw}}/\bar{\mathrm{Mn}}$
BHBP/PU-1	20.1	2.88	4.70	1.6
BHBP/PU-2	19.5	4.07	6.19	1.5
BHBP/PU-3	19.0	5.52	8.18	1.4

### 3.3. Thermal properties

**Figure 4.**
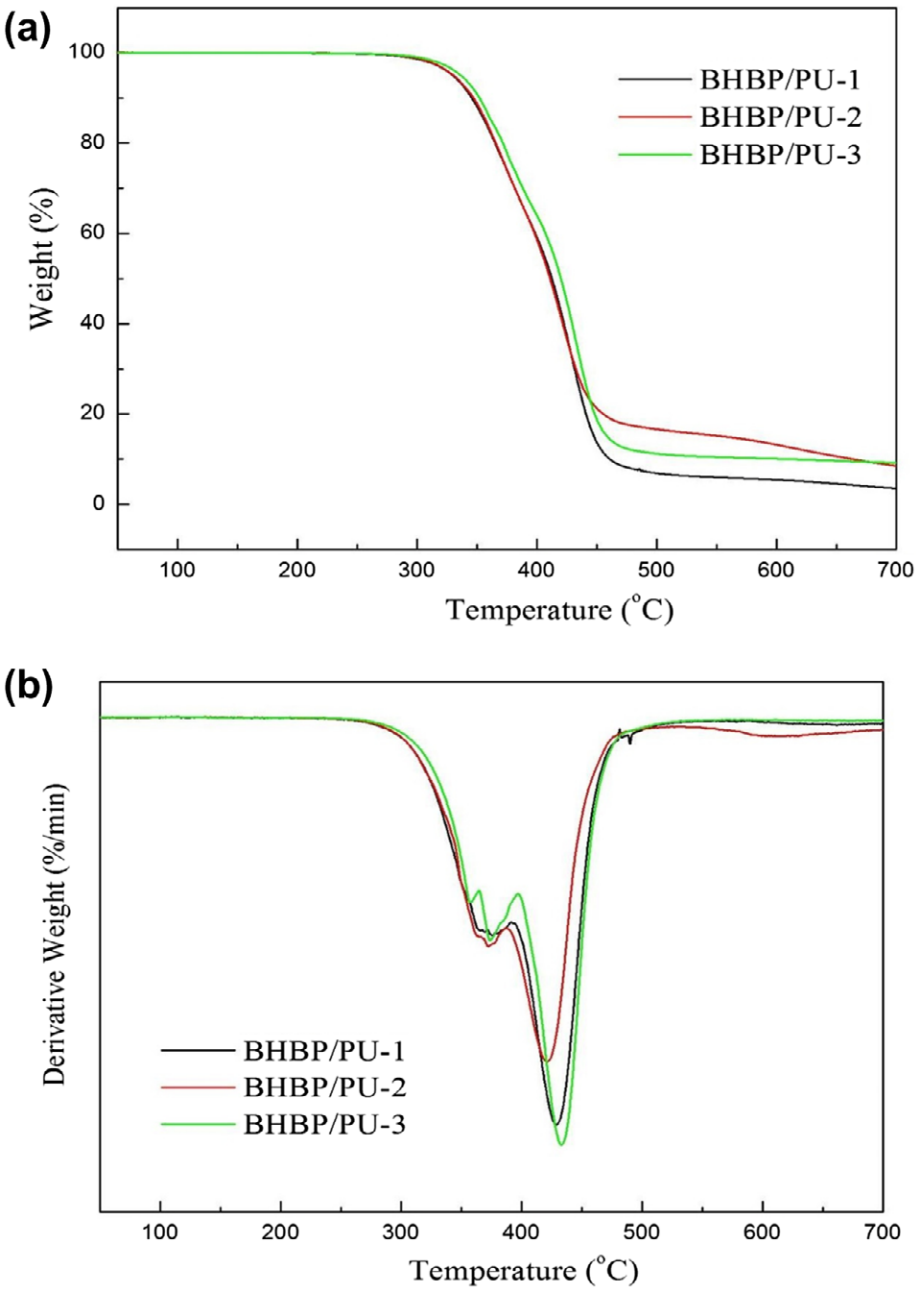
TGA and DTG curves of the BHBP/PUs.

Figure 4 presents the TGA and differential thermogravimetry (DTG) curves of the BHBP/PU samples with various chain extender contents. Two decomposition peaks were observed for each BHBP/PU sample. The decomposition peak with a lower decomposition temperature was the urethane group, whereas that with a higher decomposition temperature was the polyol. Table 3 lists the initial decomposition temperatures (i.e., onset temperature) of these polymers. The initial decomposition temperatures of BHBP/PU-1, BHBP/PU-2, and BHBP/PU-3 were 333.8, 338.1, and 341.8 °C, respectively, whereas the maximum decomposition temperatures (T_max_) were 402.9, 407.8, and 412.9 °C. BHBP/PU-1 and BHBP/PU-3 had the highest and lowest BHBP content, respectively, and the hard segment content increased as the chain extender content increased. This demonstrated that the introduction of rigid pyridine rings enhanced the thermal resistance of the PU sample. Therefore, the thermal resistance of the polymers increased as the hard segment or chain extender content increased. The pyridine content of BHBP/PU-1, BHBP/PU-2, and BHBP/PU-3 were 0.50, 0.75, and 1.00 mol, respectively. Because BHBP/PU-3 had higher pyridine content than the other two samples, more hydrogen bonds were formed within it. Therefore, hydrogen bonding was also one of the factors affecting the thermal stability of the BHBP/PU samples.

**Table 3.** Thermal properties of the BHBP/PUs.

Sample	TGA			DSC
	T_onset_(℃)	T_max_(℃)	Residue at 700℃	T_g_ (℃)
BHBP/PU-1	333.8	402.9	3.5	−55.8
BHBP/PU-2	338.1	407.8	8.5	−53.5
BHBP/PU-3	341.8	412.9	9.2	−51.0

**Figure 5.**
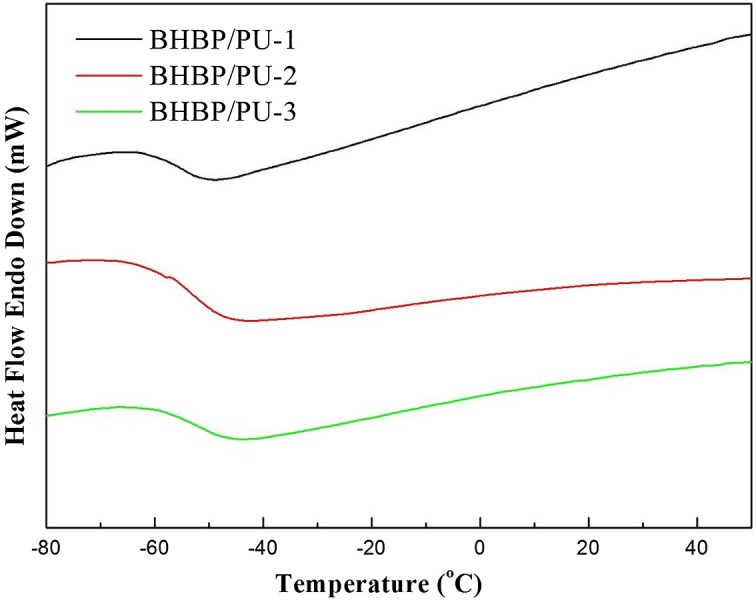
DSC thermograms of the BHBP/PUs.

Figure 5 presents the DSC curves of the three BHBP/PU samples, the features of which are summarized in Table 3. T_g_ was found to be independent of its component ratio, indicating a uniform distribution of hard and soft segments in all samples. The T_g_ of BHBP/PU-1, BHBP/PU-2, and BHBP/PU-3 was −55.8, −53.5, and −51.0 °C, respectively. The results revealed that higher BHBP content resulted in higher both T_g_ and dynamic glass transition temperature (T_gd_) of the BHBP/PU samples. This can be attributed to two factors. First, BHBP has a rigid structure. Second, the FT-IR spectra revealed numerous hydrogen bonds in the BHBP/PU samples, and these hydrogen bonds reduced the free volume of the polymer chains and inhibited their movement. The experiment confirmed that adding soft segments shifted the T_g_ of the BHBP/PU to a lower temperature, whereas adding hard segments did the opposite. These results were consistent with those of TGA, which revealed that increasing either BHBP or hard segment content enhanced the thermal stability of the PUs. In addition, Chiu et al. [20] used 2,6-pyridinedimethanol as chain extender to synthesize PU (PDM/PUs). Its Tg temperature is slightly lower than that of BHBP/PUs by about 6 °C. This may be caused because the structure of BHBP chain extender is different from that of 2,6-pyridinedimethanol. BHBP has rigid benzene ring structure, which leads less mobility of polymer chain.

## 3.4. DMA

**Figure 6.**
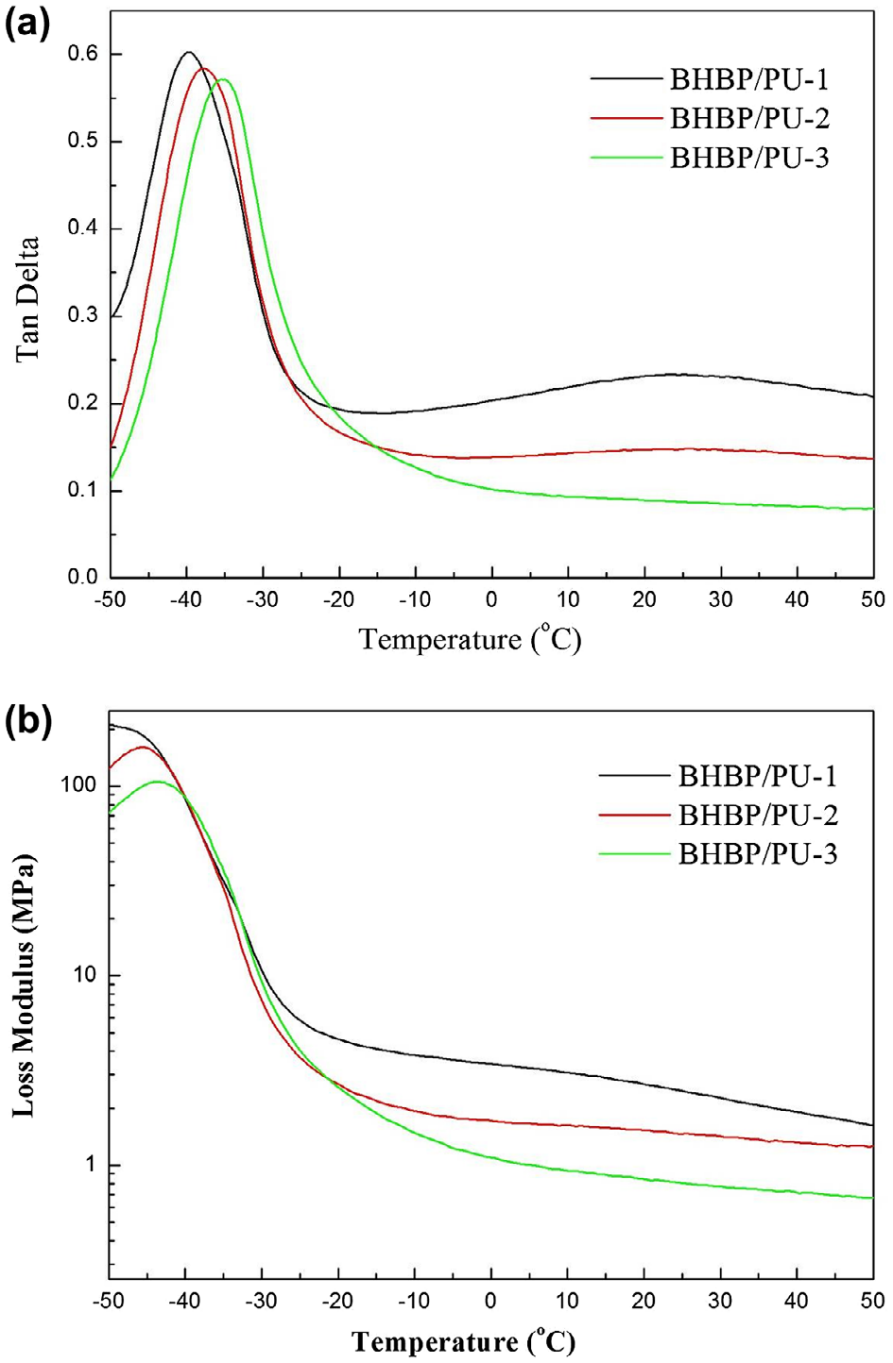
Dynamic mechanical analysis of the BHBP/PUs. (a) Tanδ, (b) loss modulus (E′).

Figure 6 shows graphs of the loss modulus (E″), storage modulus (E′), and the tan δ of the BHBP/PU samples. T_gd_ can be determined using the tan δ or E″ curve. According to the tan δ curve, BHBP/PU-1, BHBP/PU-2, and BHBP/PU-3 had a T_gd_ of −39.9, −37.7, and −35.5 °C, respectively. In this study, each T_gd_ obtained using the E″ curve was approximately 8–12 °C lower than that obtained using the tan δ curve. As the BHBP content was increased, T_gd_ increased, consistent with the aforementioned DSC results. In the BHBP/PU samples, strong hydrogen bonds were formed between the pyridine rings and N–H bonds, which inhibited segment movement and thus increased the T_gd_ of the samples. In addition, for both curves, the sample with the highest chain extender content has a higher maximum T_gd_ than those with lower chain extender content. Consequently, the BHBP/PU sample with higher hard segment content had a more elastically solid and less viscously liquid appearance than the other samples, which translated to a lower loss modulus and higher storage modulus. Because tan δ = E″/E′, BHBP/PU-3, which had the highest BHBP content, had the lowest peak value. The influence of hard segment content demonstrated that the polymers with higher chain extender content had lower viscosity (Table 4).

**Table 4.** DMA results of the BHBP/PUs.

Sample	Tgd from Tanδ (℃)	Tan δ_max_	Tgd from E″ (℃)	E″_max_ (MPa)
BHBP/PU-1	−39.9	0.6022	−51.9	216.2
BHBP/PU-2	−37.7	0.5840	−45.6	160.2
BHBP/PU-3	−35.5	0.5714	−43.5	105.9

### 3.5. Mechanical property analysis

**Figure 7.**
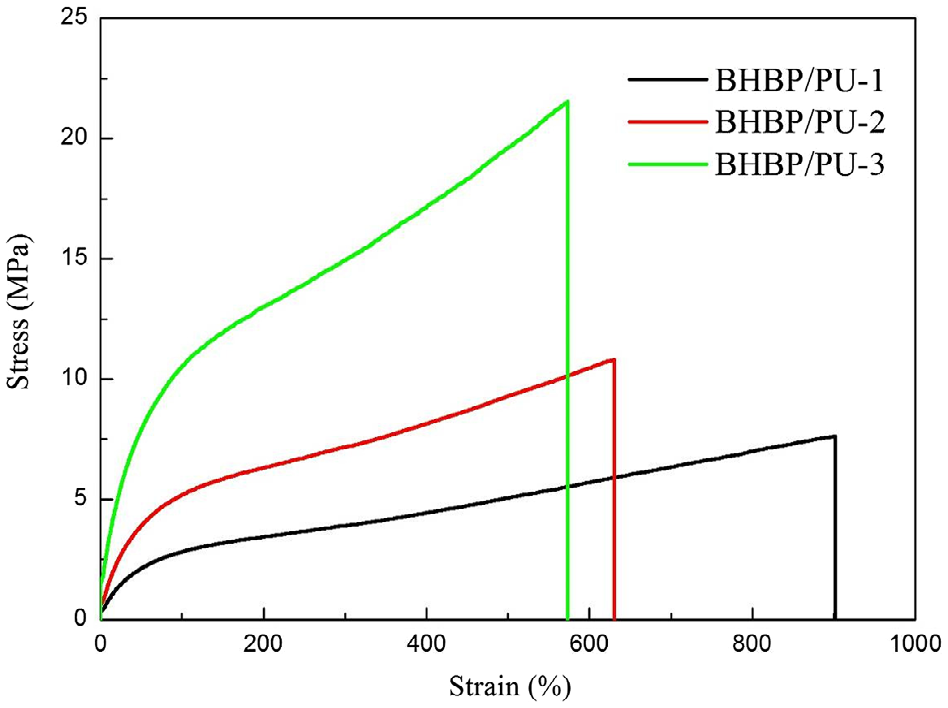
Tensile properties of the BHBP/PUs stress–strain curve.

Figure 7 presents the stress–strain curves of the BHBP/PU samples. The tensile strengths of BHBP/PU-1, BHBP/PU-2, and BHBP/PU-3 were 7.7, 10.9, and 21.6 MPa, respectively, with corresponding Young’s moduli of 0.7, 1.9, and 3.3 MPa. These results clearly demonstrated that both the tensile strength and Young’s modulus of the samples increased as the BHBP content was increased. This may be because increasing the hard segment content favored the microphase separation of the PUs. Both urethane and BHBP favored strong hydrogen bonding and intensified the microphase separation of the BHBP/PU. The elongation at break of BHBP/PU-1, BHBP/PU-2, and BHBP/PU-3 were 900, 630, and 574%, respectively. These data similarly demonstrated that increasing the hard segment content did not favor the sliding of polymer chains. However, the structure of BHBP chain extender is different from other similar chain extender with pyridine ring structure [20]. It possesses rigid benzene ring structure, so it has lower elongation at break, and this can be expected [26]. Consequently, high BHBP content decreased the elongation at break of the BHBP/PU. In addition, the strong hydrogen bonding observed between the pyridine rings and N–H bonds did not favor the sliding movements of polymer chains and further decreased the elongation at break of the polymers (Table 5).

**Table 5.** Mechanical properties of the BHBP /PUs.

Sample	Tensile strengths (MPa)	Strain at break (%)	Young’s modulus (MPa)
BHBP/PU-1	7.7	900	0.7
BHBP/PU-2	10.9	630	1.9
BHBP/PU-3	21.6	574	3.3

### 3.6. Shape recovery

**Figure 8.**
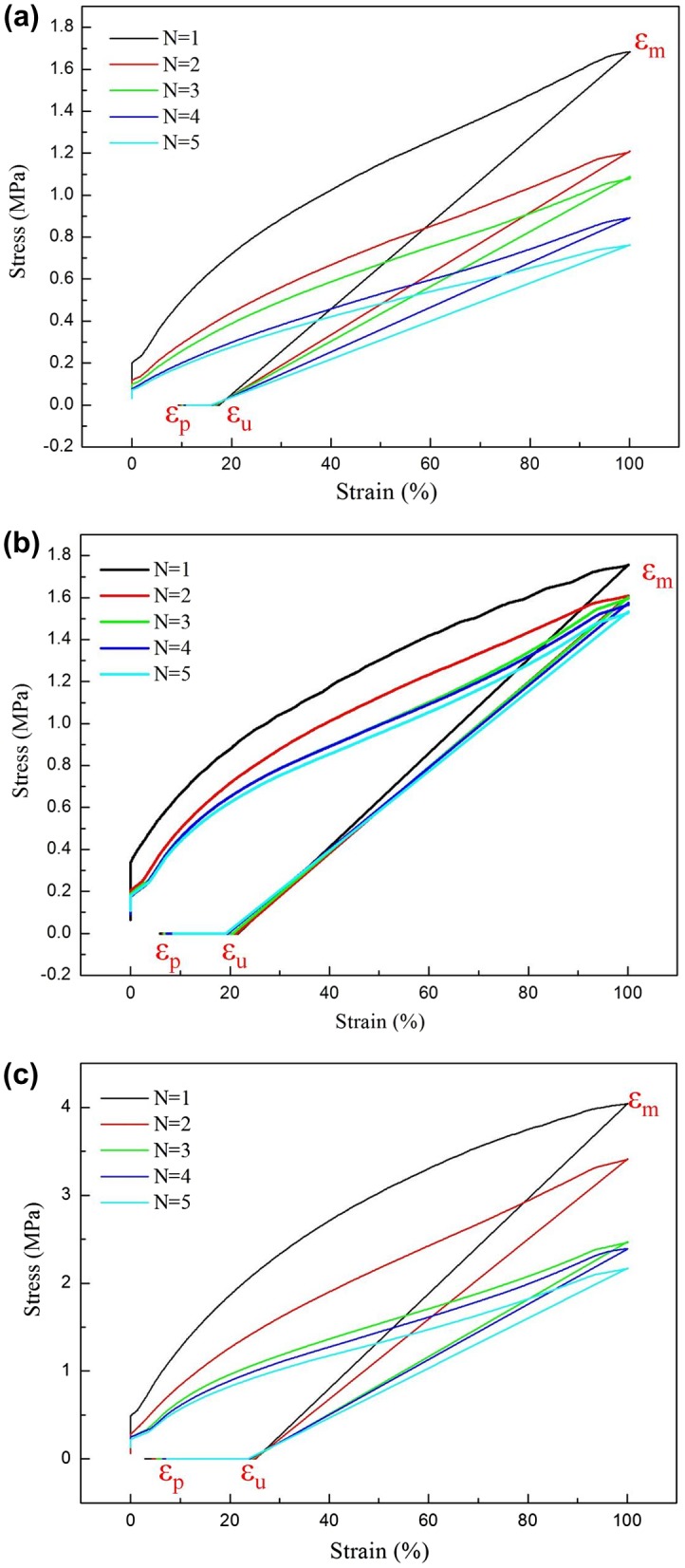
Thermomechanical cyclic behaviour of BHBP/PUs -01 (a), BHBP/PUs -02 (b), BHBP/PUs -03 (c).

Shape-memory material has the ability to restore itself to its permanent shape after deformation. All three samples in this study exhibited a heat-induced shape memory effect. The stress–strain curves in Figure 8 reveal that the three samples could be easily stretched by 100%. Therefore, all samples were extremely soft and could be elongated by 100% at 80 °C, with a higher fixity rate at room temperature because of higher glassy storage modulus. When the temperature was increased to 80 °C, the samples recovered to their original shape. The fixed strain and recovery strain could be used to calculate the shape fixity and shape recovery of the polymers [22]. BHBP/PU-3 exhibited excellent shape fixity (25.2%), whereas that of BHBP/PU-1 was only approximately 17.5% because of the presence of rigid heterocycles or a large volume of hydrogen bonds in the pyridine rings. Shape recovery improved as BHBP content was increased. For example, during the first cycle, BHBP/PU-1 and BHBP/PU-3 had 92.8 and 96.2% shape recovery, respectively. Because of the pyridine ring structure of BHBP, the effect of temperature on hydrogen bonds in the pyridine rings facilitated heat-induced shape recovery. These results demonstrated the substantial influence of pyridine content on both the shape recovery and shape fixity of the PUs. However, BHBP/PUs has higher shape fixity and lower shape recovery compared to other pyridine-containing PU [20]. This may be caused because BHBP has rigid benzene ring structure, which can reduce the flexibility of polymer chain, therefore increase shape fixity and reduce shape recovery of polymer. As the number of cycles increased, both shape recovery and shape fixity gradually decreased (Table 6).

**Table 6.** Shape recovery of the BHBP/PUs (*N* = number of thermomechanical cycle).

	Shape recovery (%)			Shape fixity (%)		
	BHBP/PU-1	BHBP/PU-2	BHBP/PU-3	BHBP/PU-1	BHBP/PU-2	BHBP/PU-3
*N* = 1	90.7	94.1	96.2	17.5	21.6	25.2
*N* = 2	90.4	93.5	95.7	17.0	21.3	25.1
*N* = 3	90.0	93.2	94.7	16.7	20.6	24.4
*N* = 4	89.5	92.8	93.6	16.2	19.5	24.1
*N* = 5	89.0	91.4	92.7	15.9	19.2	23.7

## 4. Conclusion

This study synthesized 2,6-diaminopyridine and 4-hydroxybenzaldehyde to form the pyridine-containing BHBP chain extender. PTMG and MDI were reacted to synthesize the prepolymer. Finally, the BHBP chain extender was added to the prepolymer to formulate BHBP/PU polymers. TGA revealed that the T_g_ and T_gd_ of the BHBP/PU samples increased as the BHBP content was increased, and both the tensile strength and Young’s modulus of the BHBP/PUs increased with increasing BHBP content. In addition, the BHBP/PU sample with the highest BHBP content exhibited an excellent shape recovery rate of 96.2%.

## Disclosure statement

No potential conflict of interest was reported by the authors.

## References

[1] Bayer O Das di-isocyanat-polyadditionsverfahren (polyurethane). Angewandte Chemie. 1947;59:257–272. 10.1002/(ISSN)1521-3757

[2] Woods G ICI polyurethane New York (NY): Wiley; 1990.

[3] Dombrow BA *Polyurethanes*, 2nd ed New York (NY): Reinhold Publishing Corp.; 1965.

[4] Nasreddine K, Campistron I, Albert L, et al Use of hydroxytelechelic cis-1,4-polyisoprene (HTPI) in the synthesis of polyurethanes (PUs). Part 1. Influence of molecular weight and chemical modification of HTPI on the mechanical and thermal properties of PUs. Polymer. 2005;46:6869–6877.

[5] Ratna D, Karger-Kocsis J Recent advances in shape memory polymers and composites: a review. J Mater Sci. 2008;43:254–269. 10.1007/s10853-007-2176-7

[6] Park HB, Lee YM Separation of toluene/nitrogen through segmented polyurethane and polyurethane urea membranes with different soft segments. J Membrane Sci. 2002;197:283–296. 10.1016/S0376-7388(01)00663-9

[7] Abraham GA, de Queiroz AAA, Roman JS Hydrophilic hybrid IPNs of segmented polyurethanes and copolymers of vinylpyrrolidone for applications in medicine. Biomaterials. 2001;22:1971–1985. 10.1016/S0142-9612(00)00381-1 11426875

[8] Tien YI, Wei KH Hydrogen bonding and mechanical properties in segmented montmorillonite/polyurethane nanocomposites of different hard segment ratios. Polymer. 2001;42:3213–3221. 10.1016/S0032-3861(00)00729-1

[9] Mequanint K, Sanderson R Nano-structure phosphorus-containing polyurethane dispersions: synthesis and crosslinking with melamine formaldehyde resin. Polymer. 2003;44:2631–2639. 10.1016/S0032-3861(03)00154-X

[10] Gugliuzza A, Clarizia G, Golemme G, et al New breathable and waterproof coatings for textiles: effect of an aliphatic polyurethane on the formation of PEEK–WC porous membranes. Eur Polym J. 2002;38:235–242. 10.1016/S0014-3057(01)00186-0

[11] Xie T, Rousseau IA Facile tailoring of thermal transition temperatures of epoxy shape memory polymers. Polymer. 2009;50:1852–1856. 10.1016/j.polymer.2009.02.035

[12] Zhang SZJ, Yu T, Govender HY, et al A novel supramolecular shape memory material based on partial α-CD–PEG inclusion complex. Polymer. 2008;49:3205–3210. 10.1016/j.polymer.2008.05.030

[13] Lendlein A, Jiang HY, Jünger O, et al Light-induced shape-memory polymers. Nature. 2005;434:879–882. 10.1038/nature03496 15829960

[14] Chen S, Hu J, Yuen CW, et al Novel moisturesensitive shape memory polyurethanes containing pyridine moieties. Polymer. 2009;50:4424–4428. 10.1016/j.polymer.2009.07.031

[15] Chen SJ, Hu JL, Chen SG, et al Study on the structure and morphology of supramolecular shape memory polyurethane containing pyridine moieties. Smart Mater Struct. 2011;20(065003):9 DOI: 10.1088/0964-1726/20/6/065003.

[16] Lendlein A, Kelch S Shape-Memory Polymers. Angew Chem Int Ed. 2002;41:2034–2057. 10.1002/1521-3773(20020617)41:12&lt;2034::AID-ANIE2034&gt;3.0.CO;2-M 19746597

[17] Liu C, Qin H, Mather PT Review of progress in shape-memory polymers. J Mater Chem. 2007;17:1543–1558. 10.1039/b615954 k

[18] Liu C, Mather PT Thermomechanical characterization of a tailored series of shape memory polymers. J Appl Med Polym. 2002;6:47–52.

[19] Yakacki CM, Shandas R, Safranski D, et al Strong, tailored, biocompatible shape-memory polymer networks. Adv Func Mater. 2008;18:2428–2435. 10.1002/adfm.v18:16 PMC2714647 19633727

[20] Chiu SH, Wu CL, Tsou CY, et al Study of the synthesis and properties of polyurethane containing pyridyl units for shape memory. Polymer Bulletin. 2016;73:1303–1320. 10.1007/s00289-015-1548-4

[21] Chen S, Hu J, Zhuo H, et al Study on the thermal-induced shape memory effect of pyridine containing supramolecular polyurethane. Polymer. 2010;51:240–248. 10.1016/j.polymer.2009.11.034

[22] Chen S, Hu J, Yuen CW, et al Supramolecular polyurethane networks containing pyridine moieties for shape memory materials. Mater Lett. 2009;63:1462–1464. 10.1016/j.matlet.2009.03.028

[23] Oprea S, Potolinca VO, Varganici CD Synthesis and properties of polyurethane urea with pyridine-2,6-dicarboxamide moieties in their structure. RSC Adv. 2016;6:106904–106913. DOI:10.1039/C6RA23660J.

[24] Potolinca VO, Buruiana E, Oprea S Dielectric behavior of polyurethane and polyurethane-urea elastomers with pyridine moieties in the main chain. J Polym Res. 2013;20:237–242. 10.1007/s10965-013-0237-y

[25] Reddy KR, Raghu AV, Jeong HM, et al Synthesis and characterization of pyridinebased polyurethanes. Des Monomers Polym. 2009;12:109–118. 10.1163/156855509X412054

[26] Liaw DJ The relative physical and thermal properties of polyurethane elastomers: effect of chain extenders of bisphenols, diisocyanate, and polyol structures. J Appl Polym Sci. 1997;66:89–104.

